# A Multicenter Cohort Study on the Adverse Effects Evaluation After Messenger RNA COVID-19 Vaccination Among Pregnant Healthcare Employees in Penang General Hospitals

**DOI:** 10.3389/fpubh.2022.876966

**Published:** 2022-05-23

**Authors:** Ann Lisa Arulappen, Monica Danial, Gaiyathri Shanmugam, Joo Thye Cheng, Mairin Dulasi, Ting Soo Chow

**Affiliations:** ^1^Department of Pharmacy, Seberang Jaya Hospital, Ministry of Health, Permatang Pauh, Malaysia; ^2^Clinical Research Center, Seberang Jaya Hospital, Ministry of Health, Permatang Pauh, Malaysia; ^3^Department of Medicine, Seberang Jaya Hospital, Ministry of Health, Permatang Pauh, Malaysia; ^4^Department of Obstetrics & Gynecology, Seberang Jaya Hospital, Ministry of Health, Permatang Pauh, Malaysia; ^5^Department of Medicine, Penang Hospital, Ministry of Health, George Town, Malaysia

**Keywords:** COVID-19 vaccine, mRNA vaccine, pregnancy outcome, adverse effects post vaccination, neonatal outcomes, safety of mRNA vaccine

## Abstract

**Introduction:**

The year 2020 saw the emergence of novel severe acute respiratory syndrome coronavirus 2 (SARS-CoV-2), which became a great threat to public health worldwide. The exponential spread of the disease with millions of lives lost worldwide saw the emergence of an accelerated vaccine development with emergency approval from well-known regulatory bodies such as the US Food and Drug Administration, followed by widespread vaccine deployment despite a paucity in safety profile data. This issue becomes even more pronounced when it involves expectant mothers considering the possible undesirable effect toward the unborn child.

**Method:**

This was a retrospective cohort study which was conducted at six general hospitals in the state of Penang, Malaysia. All the pregnant employees who have consented to take the mRNA COVID-19 vaccine and participate in this study were monitored from the time of their first vaccination and up to 28 days after they delivered their babies.

**Results:**

All the participants had adequate maximum vertical pocket (MVP) and no obvious anomalies or detection of intrauterine growth restriction (IUGR) were detected during the second trimester. However, one subject was reported to have miscarried during the second trimester. The reported mean neonate birth weight was 3.0 kg with the mean Apgar score of 8.8 and 9.8 at 1 and 5 min, respectively. Approximately seven (5.8%) neonates were reported to be small for their gestational age. Another three (2.5%) neonates were reported to have anomalies.

**Conclusion:**

As a whole, the inference that can be made from this study is that mRNA COVID-19 vaccine appears to be safe in pregnant women regardless of the trimester as the findings did not show obvious safety warning signs.

## Introduction

The year 2020 saw the emergence of novel severe acute respiratory syndrome coronavirus 2 (SARS-CoV-2), which became a great threat to public health worldwide. The epicenter of the outbreak was reported in Wuhan City, China in December 2019 (1). Subsequently, it spread like wildfire to the rest of the world and became a nightmare scenario as the number of deaths in conjunction to this infection increased drastically. Unfortunately, the effective treatment and definitive cure is yet to be discovered. The exponential spread of the disease with millions of lives lost worldwide saw the emergence of an accelerated vaccine development with emergency approval from well-known regulatory bodies such as the US Food and Drug Administration, followed by widespread vaccine deployment despite a paucity in safety profile data. Amongst pregnant women, data was even scarcer as they were not represented in the original clinical trials, especially when the expectant women have to consider the possible undesirable effect toward the unborn child as well.

Literature has revealed that pregnant women with severe COVID-19 infection are at increased risk for preterm birth and even pregnancy loss (2). As a whole, when compared to non-pregnant women, those who are pregnant are at higher risk of acquiring multiple complications through COVID-19 infection which may consequently result in greater mortality rate (3). Therefore, it is crucial to prevent COVID-19 infection from spreading through mass vaccination to attain herd immunity. In fact, medical bodies all around the world including the WHO, the Centers for Disease Control and Prevention (CDC), and Advisory Committee on Immunization Practices (ACIP), in collaboration with the American College of Obstetricians and Gynecologists and the American Academy of Pediatrics, have issued guidance recommending vaccines for pregnant women, especially those at highest risk (4–6).

Multiple studies have reported on the potential benefits of mRNA vaccines over live attenuated virus vaccines, inactivated or subunit vaccines, and DNA-based vaccines (7). In fact, they are manufactured by many registered pharmaceutical companies such as Astra Zeneca, Pfizer, etc. and are distributed worldwide in conjunction with the mass vaccination program. In Malaysia, there are two types of vaccines available, namely Pfizer/BioNTech (BNT162b2) and Oxford/AstraZeneca (AZD1222). However, only Pfizer is approved to be used in pregnant women by the National Pharmaceutical Regulatory Agency (NPRA), Malaysia, which is the mRNA COVID-19 (8). The safety profile for mRNA COVID-19 vaccine is yet to be established (9).

Therefore, it is necessary to establish the safety data in real time through rigorous proactive data collection to record both vaccine-related symptoms as well as obstetric outcomes which will advance current understanding on the symptoms encountered. Additionally, it provides further guidance for pregnant people to get vaccinated and also for the national policy holders during policy formulation.

## Method

### Study Design and Setting

This was a retrospective cohort study which was conducted at six general hospitals in the state of Penang, Malaysia. These hospitals have the capacity to serve a population of 1.77 million approximately as sourced from the Department of Statistics Malaysia (10). Ethical board approval by MREC was obtained prior to the initiation of this study (NMRR-21-496-59218).

### Study Population

The recruited subjects were identified from the mass vaccination registry maintained at each hospital. All the pregnant employees who consented to take the mRNA COVID-19 vaccine and participate in this study were monitored from the time of their first vaccination and up to 28 days after they delivered their babies. They were contacted about 10 times throughout this study and each session lasted about 10 min. The vaccinated pregnant employees who refused to give consent to participate in this study were excluded.

### Data Collection

A separate datasheet was used in Microsoft Excel to aid the analysis process. The data collected includes patient demographics, antenatal and post-natal history, any reported side effects post vaccination, fetal growth or development by serial ultrasound scans, birth outcomes, and the infants' development monitored up to 28 days of life as per the recommendation by Centers for Disease Control and Prevention (CDC) (11), which was adopted as a policy by our health facility.

### Statistical Analysis

Statistical analysis was performed using SPSS (version 23; SPSS, Inc., Chicago, 1L) statistical software. Descriptive analysis was used to summarize the collected data into median [interquartile ranges (IQR)], mean ± standard deviation (SD), or frequencies (%) as appropriate.

### Outcome Measurement

This study was performed to describe the possible adverse effects associated with post-mRNA COVID-19 vaccination among the pregnant employees in government health facilities and their respective obstetric outcomes including the neonates up to 28 days of life.

## Results

This study was initiated once the Ministry of Health, Malaysia encouraged pregnant health care workers to be vaccinated and this study included the very first batch of volunteers to be vaccinated from March 2021 onwards. These subjects who consented to participate in this study were followed until their respective delivery dates. The sample size was undeniably small, but it represented 15% of the total Malaysian population. Additionally, no similar study was conducted elsewhere in Asia and so this study would provide the baseline data among this population group. Under the national procurement policy, only Pfizer was used with multiple batches of vaccines. A total of 121 pregnant healthcare employees participated in this study with the highest percentage of 57% (*n* = 69) from the 30–39 years of age category and up to 71.9% (*n* = 87) from the second trimester when the subjects received the first dose of the mRNA COVID-19 vaccine. Although the recommended dosing interval between the first and second dose was 3 weeks, the median (IQR) interval in this study was found to be 21 weeks as the Ministry of Health did not impose any restrictions on the permitted dosing gap between two doses. The majority of them, 91% (*n* = 109), received the mRNA COVID-19 vaccine within the recommended dosing interval. However, only four out of 121 subjects tested positive for COVID-19 infection post-mRNA vaccination despite all four individuals being vaccinated within the recommended dosing interval and having no comorbidities. All of them were hospitalized and were categorized as non-severe COVID-19 (12). All of them had a full recovery without any complications. The overall demographics and post-vaccination characteristics after mRNA COVID-19 vaccination by pregnant healthcare employees in general hospitals in Penang is depicted in Table 1. All the lab investigations pertaining to the blood parameters taken during subjects' antenatal visits were reported to be normal.

**Table 1:** Demographics and post vaccination characteristics post messenger RNA (mRNA) COVID-19 vaccination by pregnant healthcare personnel in general hospitals in Penang, Malaysia (*n* = 121).

**Demographics characteristics**	***n* (%)**
**Age, median (IQR), years**	30 (5)
20–29 years	45 (37.2)
30–39 years	69 (57.0)
40–49 years	7 (5.8)
**Ethnicity**	
Malay	101 (83.5)
Chinese	12 (9.9)
Indian	7 (5.8)
Murut	1 (0.8)
**Position**	
Doctor	37 (30.6)
Nurse	54 (44.6)
Pharmacist	8 (6.6)
Others	22 (18.2)
**Specialty**	
Anesthesiology	16 (13.2)
Medical	16 (13.2)
OG	13 (10.7)
General surgery	10 (8.3)
Orthopedic	10 (8.3)
Pediatric	10 (8.3)
Others	46 (38.0)
Stage of pregnancy during vaccination, mean (SD), weeks	22 (6.7)
First trimester, 1–13 weeks of pregnancy	3 (2.5)
Second trimester, 14–27 weeks of pregnancy	87 (71.9)
Third trimester, 28 and onwards weeks of pregnancy	31 (25.6)
**Number of children currently alive**	
No children	34 (28.1)
1–2 children	73 (60.3)
≥3 children	14 (11.6)
Interval between first and second vaccination, median (IQR), weeks	21 (0)
**COVID-19 status post vaccination**	
Negative for COVID-19	117 (96.7)
Positive for COVID-19	4 (3.3)

The majority of the study participants, ~74.2% (*n* = 92), had no previously reported significant antenatal history (ANC) history and another 60.6% (*n* = 77) had no actively reported ANC history for current pregnancies. Additionally, a majority of them, 91.1% (*n* = 113), had no reported known allergies to medications. It is further tabulated in Table 2.

**Table 2:** Previous and current antenatal care (ANC) history and reported medication allergies of pregnant healthcare personnel in general hospitals in Penang, Malaysia (*n* = 121).

	***n* (%)**
**Previous antenatal care (ANC) history**	
Gestational diabetes mellitus	18 (14.5)
Anemia	10 (8.1)
Hyperthyroidism	2 (1.6)
Bronchial asthma	1 (0.8)
Impending eclampsia	1 (0.8)
No previously reported ANC history	92 (74.2)
**Current significant antenatal care (ANC) history**	
Gestational diabetes mellitus	31 (24.4)
Anemia	16 (12.6)
Bronchial asthma	1 (0.8)
Hyperthyroidism	1 (0.8)
Pelvic inflammatory disease	1 (0.8)
No currently reported ANC history	77 (60.6)
**Medication allergies**	
Diclofenac sodium	2 (1.6)
Erythromycin ethylsuccinate	2 (1.6)
Carbimazole	1 (0.8)
Hyoscine	1 (0.8)
Ibuprofen	1 (0.8)
Metoclopramide	1 (0.8)
Paracetamol	1 (0.8)
Penicillin	1 (0.8)
Iron sucrose	1 (0.8)
No allergies	113 (91.1)

### Possible Adverse Effects Associated With Post mRNA COVID-19 Vaccination Among the Pregnant Healthcare Women

**Figure 1:**
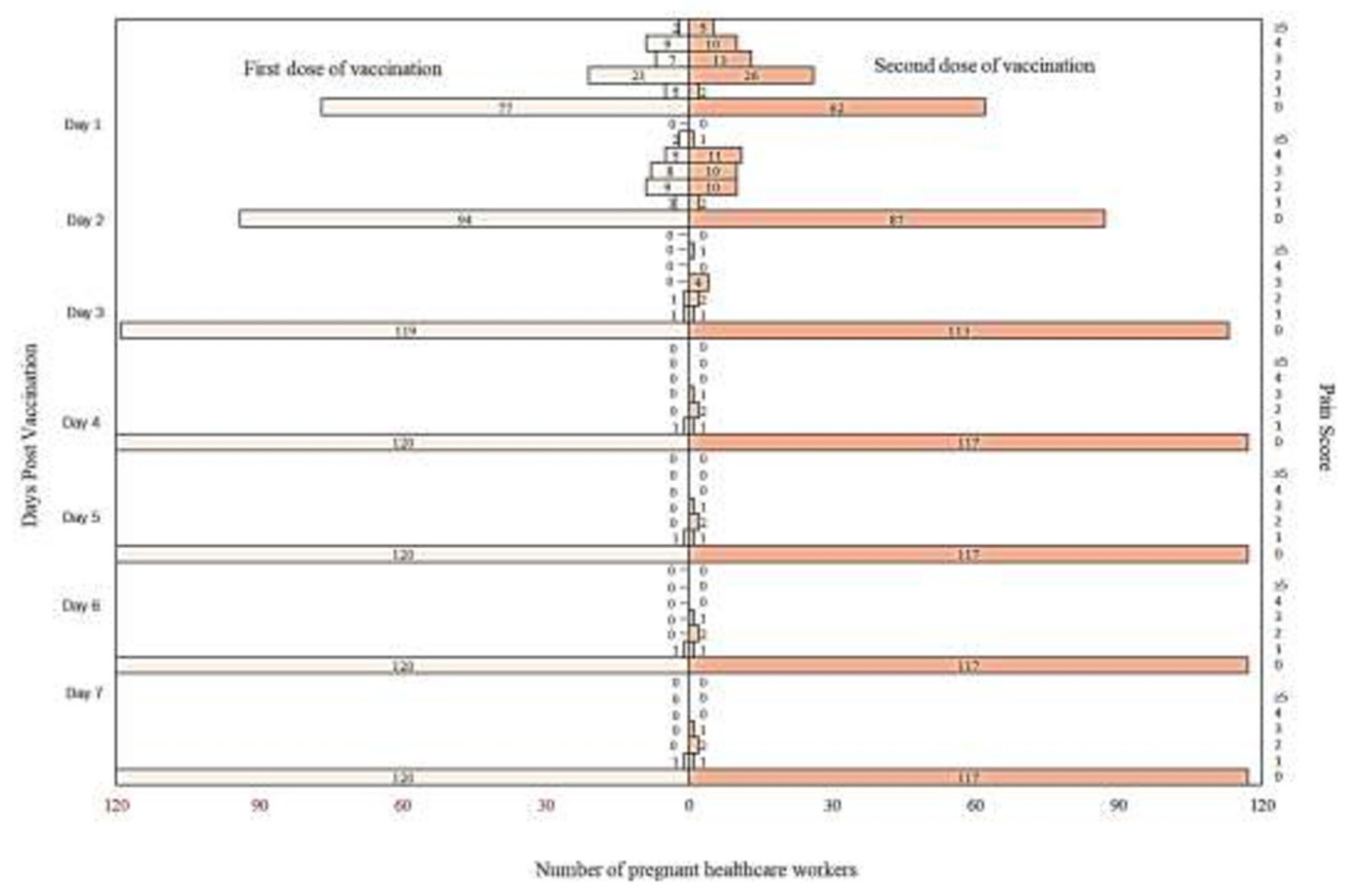
Self-reported pain score post messenger RNA (mRNA) COVID-19 vaccination by pregnant healthcare personnel in general hospitals in Penang, Malaysia (*n* = 121).

**Figure 2:**
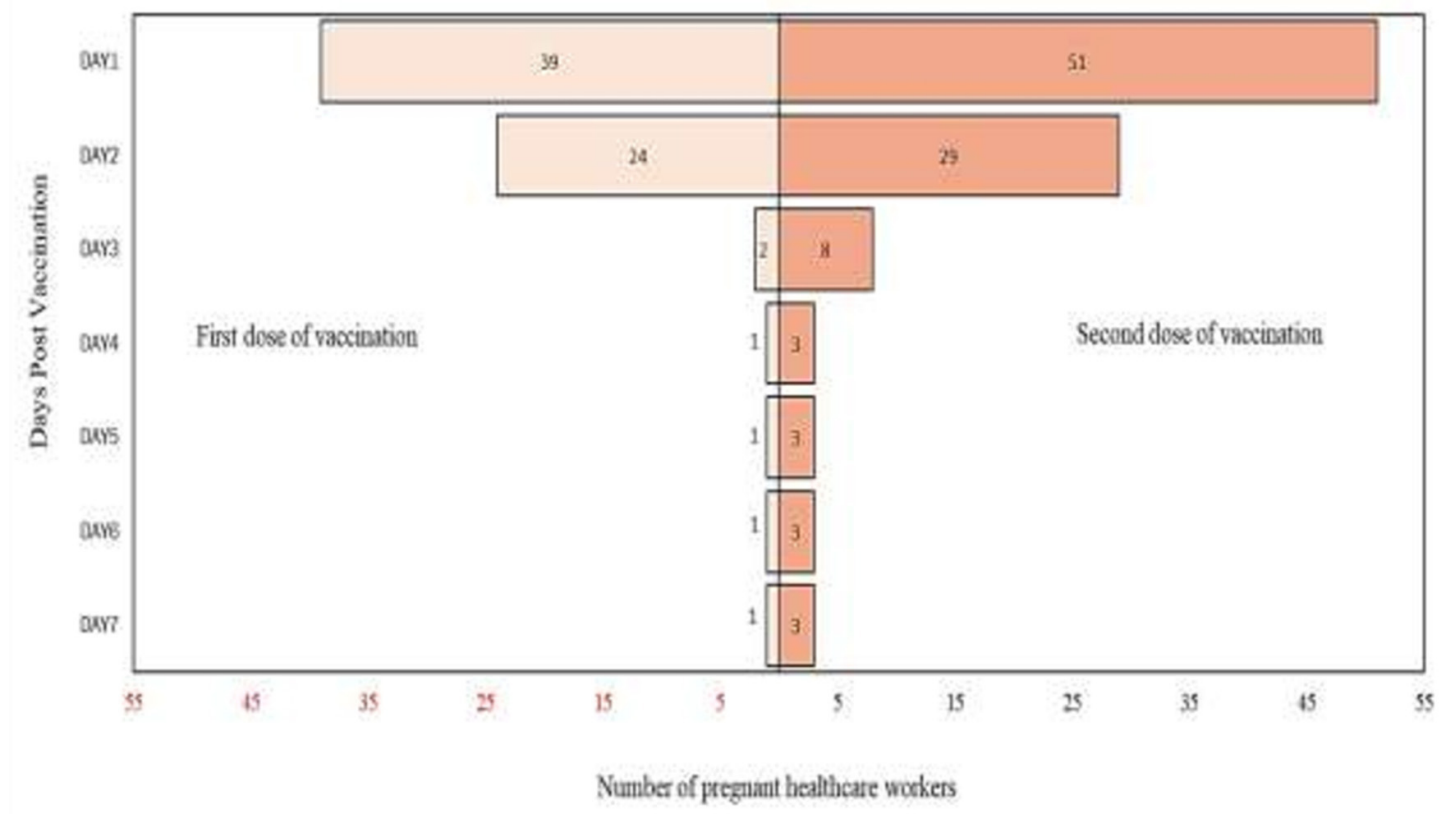
Body ache reported post messenger RNA (mRNA) COVID-19 vaccination by pregnant healthcare personnel in general hospitals in Penang, Malaysia (*n* = 121).

**Figure 3:**
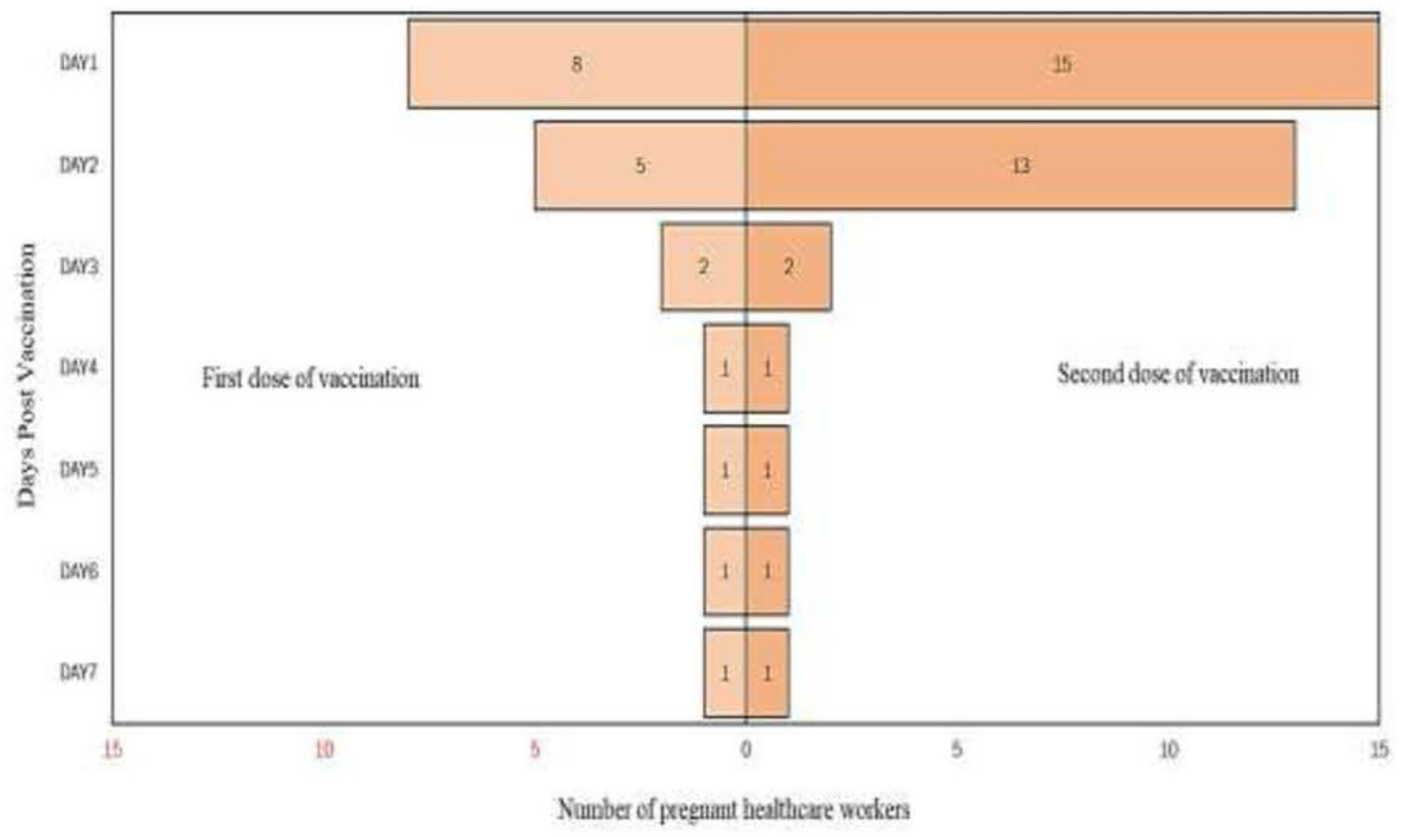
Headache reported post messenger RNA (mRNA) COVID-19 vaccination by pregnant healthcare personnel in general hospitals in Penang, Malaysia (*n* = 121).

**Figure 4:**
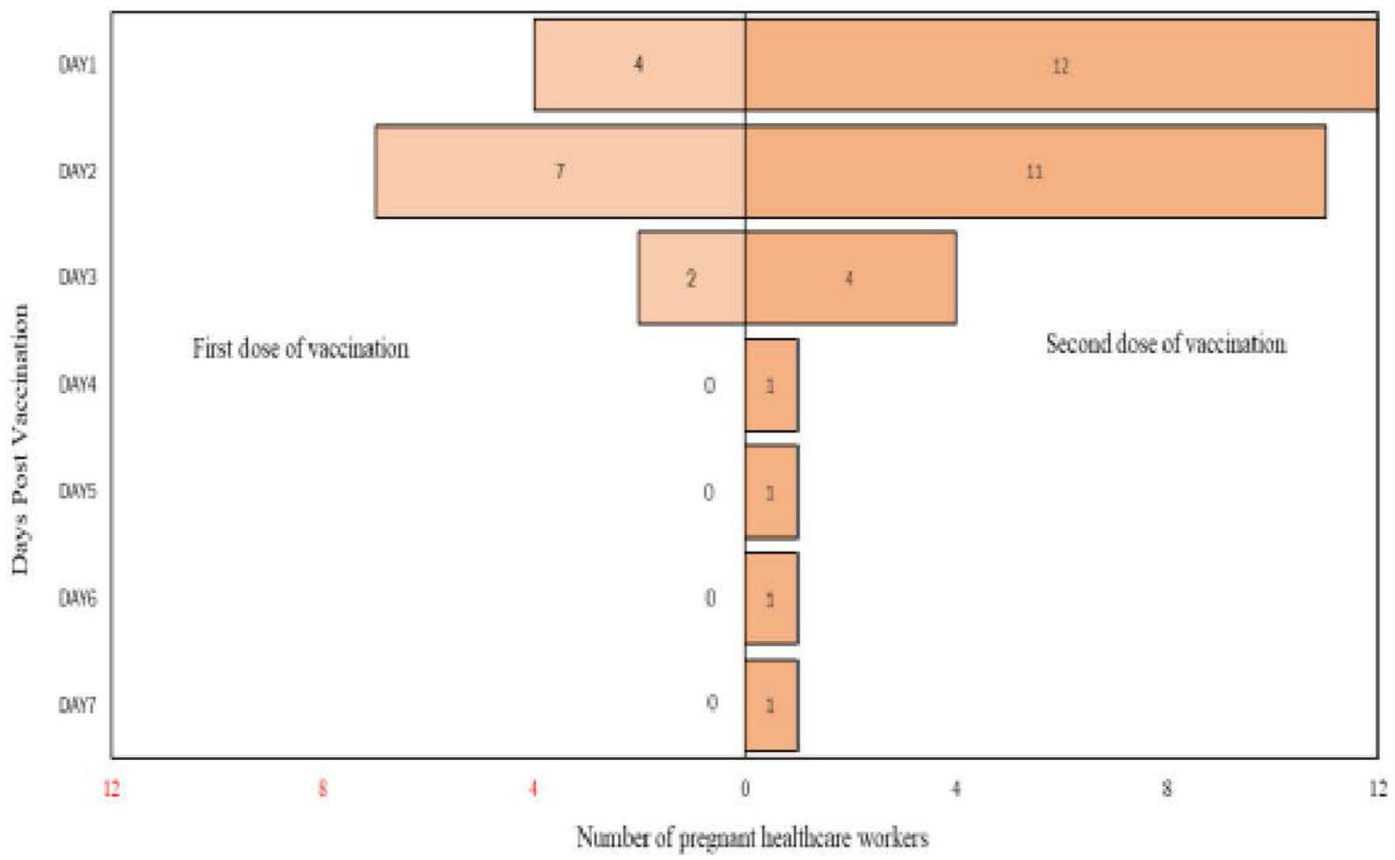
Chills reported post messenger RNA (mRNA) COVID-19 vaccination by pregnant healthcare personnel in general hospitals in Penang, Malaysia (*n* = 121).

**Figure 5:**
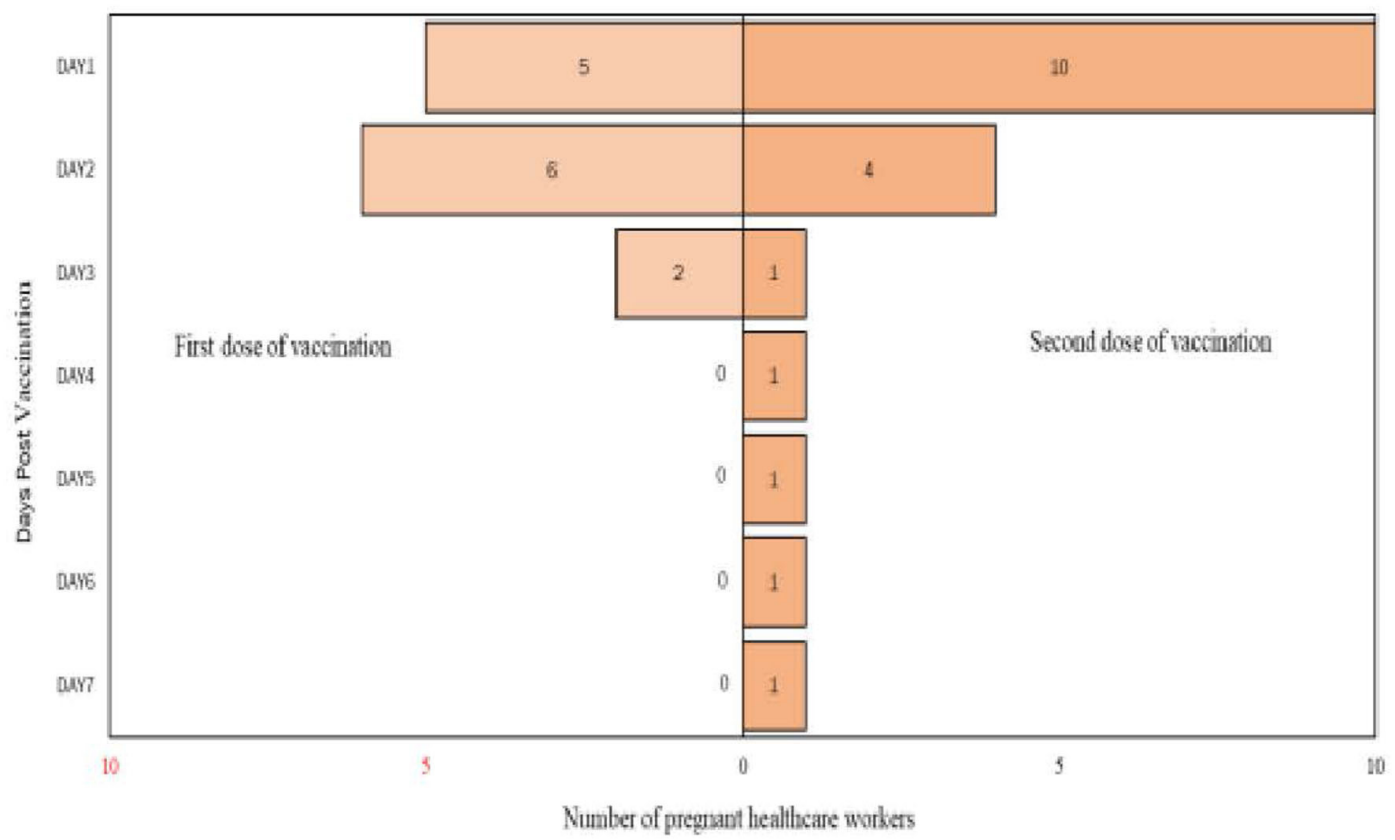
Shivering reported post messenger RNA (mRNA) COVID-19 vaccination by pregnant healthcare personnel in general hospitals in Penang, Malaysia (*n* = 121).

**Figure 6:**
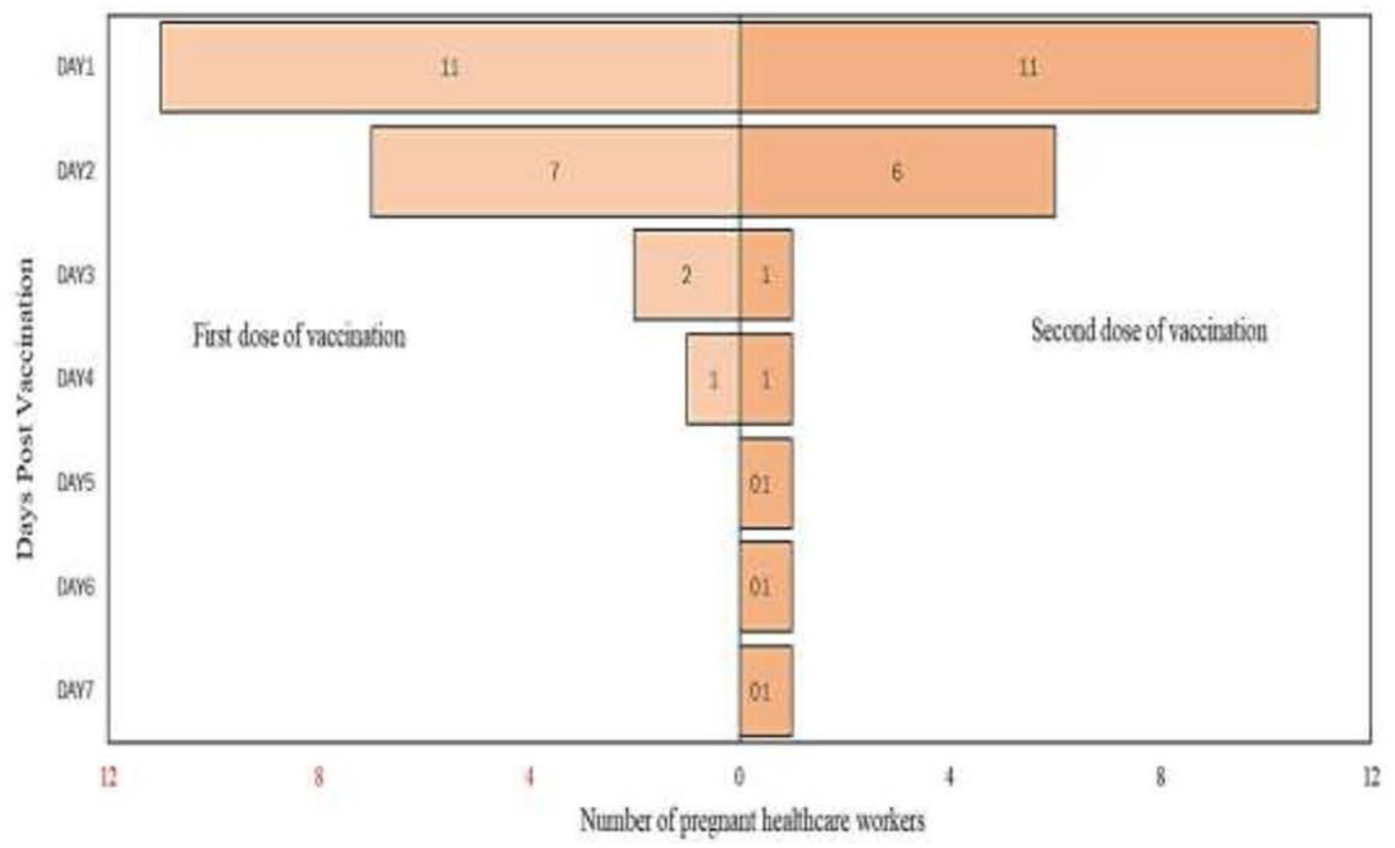
Fatigue reported post messenger RNA (mRNA) COVID-19 vaccination by pregnant healthcare personnel in general hospitals in Penang, Malaysia (*n* = 121).

From the analysis, it is found that there are more participants who complained of pain up to day 7 after the second dose of vaccination compared to post the first dose of vaccination. Detailed analysis on the self-reported pain score post mRNA COVID-19 vaccination by pregnant healthcare employees is shown in Figure 1. The most commonly reported symptoms post mRNA COVID-19 vaccination by pregnant employees were body ache, headache, chills, shivering, and fatigue which is further illustrated from Figures 2–6.

### Respective Obstetric Outcomes of Pregnant Healthcare Women Post mRNA COVID-19 Vaccination

The pregnancy monitoring is presented in Table 3. The mean estimated birth weight was 0.6 and 2.3 kg, while the mean fetal heart rate was 148 and 149 bpm in the second and third trimester, respectively. All the participants had adequate MVP with no obvious anomalies or IUGR detected during the second semester.

**Table 3:** Pregnancy monitoring outcome post messenger RNA (mRNA) COVID-19 vaccination of pregnant healthcare personnel in general hospitals in Penang, Malaysia.

**Parameters observed during pregnancy**	***n* (%)**
Second trimester (14–27 weeks of pregnancy) (*n* = 121)	
Estimated birth weight, mean (SD), kg	0.6 (0.12)
Fetal heart rate, mean (SD), beats per minute (rpm)	148 (6.3)
**Maximum vertical pocket (MVP)**	
Normal	121 (100)
Abnormal (oligohydramnios or polyhydramnios)	0
**Detection of anomalies**	
No	121 (100)
Yes	0
**Detection of intrauterine growth restriction (IUGR)**	
No	121 (100)
Yes	0
**Third trimester (28 and onwards weeks of pregnancy;** ***n*** **=** **120)**	
Estimated birth weight, mean (SD), kg	2.3 (0.52)
Fetal heart rate, mean (SD), rate per minute (rpm)	149 (6.0)
**Maximum vertical pocket (MVP)**	
Normal	120 (100)
Abnormal (oligohydramnios or polyhydramnios)	0(0)
**Detection of anomalies**	
No	120 (100)
Yes	0(0)
**Detection of intrauterine growth restriction (IUGR)**	
No	120 (100)
Yes	0 (0)
**Delivery week, median (IQR), weeks (days)**	38 (4)
Method of delivery	
Spontaneous vaginal delivery (SVD)	83 (68.6)
Cesarean section (C-sec)	37 (31.4)

In term of neonates monitoring as shown in Table 4, the reported mean neonate birth weight was 3.0 kg with the mean Apgar score of 8.8 and 9.8 at 1 and 5 min, respectively. Approximately seven (5.8%) neonates were reported to be small for their gestational age (SGA). Compared with the historical data in Malaysia which was extracted from the National Obstetrics Registry (NOR), the overall prevalence of SGA in Malaysian tertiary hospitals was 17.2%. Hence, this could possibly exclude the fact that injecting mRNA COVID-19 vaccine during pregnancy may cause neonates to be smaller for the gestational age (13). Another three (2.5%) neonates were reported to have anomalies, namely bilateral clubfoot minor, glucose-6-phosphate dehydrogenase (G6PD) deficiency, and also sacral dimple. However, only clubfeet is among the common anomalies in Malaysia, which is up to 9.1% based on a population study (14). In regars to those who had congenital anomalies, none received mRNA COVID-19 vaccine during the first trimester and the pattern of anomalies observed were vast. Out of 120 neonates, two (1.7%) of them had complaints within 4 weeks of life including constipation and another one was yet to be discharged from the hospital in view of requiring oxygen therapy (premature neonate diagnosed as Respiratory Distress Syndrome). There are infants noted to have reduction in Apgar score (minimum 7) post 10 min. Additionally, 14 of them were premature neonates but 27 (22.5%) of them were admitted into Neonatal Intensive Care Unit (NICU) for close monitoring. About 8% of babies are born preterm in Malaysia (15). However, only 12 out of 27 neonates were intubated, mostly due to respiratory distress. About 14 (11.7%) of them were treated for neonatal infection post-delivery, which was mostly empirically for congenital pneumonia with the mean antibiotic, duration of 5 days. Remarkably, the incidence of early onset sepsis (<3 days) was 0.62 (95% CI 0.45–0.82) per 1,000 live births in Asia. Unfortunately, no specific study focusing on neonatal infection post-delivery was conducted in Malaysia (16). Nevertheless, neonatal death was not reported at the point of phone interview with the mothers.

**Table 4:** Neonates monitoring outcome post messenger RNA (mRNA) COVID-19 vaccination of pregnant healthcare personnel in general hospitals in Penang, Malaysia (*n* = 120).

**Parameters observed in neonates**	***n* (%)**
Birth weight, mean (SD), kg	3.0 (0.46)
Mean Apgar score at 1 min, mean (SD)	8.8 (0.80)
Mean Apgar score at 5 min, mean (SD)	9.8 (0.47)
**Small for gestational age**	
No	113 (94.2)
Yes	7 (5.8)
**Presence of anomalies**	
No	117 (97.5)
Yes	3 (2.5)
**Jaundice requiring phototherapy**	
No	17 (14.2)
Yes	103 (85.8)
**Significant complaints within 4 weeks of life**	
No	118 (98.3)
Yes	2 (1.7)
**Preterm birth (≤37 weeks)**	
No	106 (88.3)
Yes	14 (11.7)
**Admission to NICU**	
No	93 (77.5)
Yes	27 (22.5)
**Neonatal infection**	
No	106 (88.3)
Yes	14 (11.7)
**Neonatal death or stillbirth**	
No	100 (0)
Yes	0 (0)

## Discussion

The study population in this study consists of participants who received mRNA COVID-19 vaccine in all the trimesters of pregnancy. The total documented adverse effects associated to the mRNA COVID-19 vaccine in Penang which was reported to the National Pharmaceutical Regulatory Agency (NPRA) till December 2021 were 142 reports. Out of the total number, only four reports were associated with pregnant individuals who were enrolled in this study. The first reported adverse effect post mRNA COVID-19 vaccine was severe nausea and vomiting (21 weeks pregnant at the time of complaint) which required admission for close monitoring. Symptoms resolved gradually within 2 days after the administration of Veloxin Tablet. The second report was related to chest tightness accompanied with shortness of breath (29 weeks pregnant at the time of complaint) which also required hospitalized for close monitoring. No specific treatment was initiated as electrocardiogram (ECG) showed sinus rhythm and symptoms resolved spontaneously thereafter. The third reported adverse effect was generalized body rashes involving maculo-papular rash (exanthem) over all limbs and upper trunk of the body (27 weeks pregnant at the time of complaint). Symptoms were controlled with regular application of aqueous and steroid-containing cream. This participant required only one dose of Hydrocortisone intravenously. The fourth report was quite devastating, involving severe vaginal bleeding which consequently led to miscarriage (22 weeks pregnant at the time of complaint). This participant did not receive the mRNA COVID-19 vaccine during the first trimester. A definite conclusion failed to be made that this miscarriage was attributed to the mRNA COVID-19 vaccine. In comparison of the risk of miscarriage post mRNA COVID-19 vaccine, it was found to be ~0.8% in this study vs. up to 2.6% in the other study (9).

Looking into the neonatal outcomes, the studied parameters were small for gestational age, presence of congenital anomalies, preterm birth (<37 week), and also neonatal death. For all the parameters, the calculated incidences were almost similar to the published incidences in the other peer reviewed literature which was conducted prior to the COVID-19 pandemic except small for gestational age (17–24). The incidence of small for gestational age in this study seems to be higher, at 5.8% compared to the incidences published in other studies mentioned above.

However, the top five commonly reported symptoms post mRNA COVID-19 vaccine among the pregnant women were similar to what was reported *via* V-safe Pregnancy Registry (9).

## Limitations

There are two limitations encountered in this study. Firstly, the sample size is comparatively small as certain congenital anomalies are considered rare. Therefore, occurrence of such rare disorders requires large-scale recruitment. Secondly, the neonates are monitored up to 28 days only. The adverse effects presented beyond this period remain undocumented, especially certain chronic conditions which could possibly be noticed or known in the later stage of their lives.

## Conclusion

As a whole, the inference that can be made from this study is mRNA COVID-19 vaccine appears to be safe in pregnant women regardless of the trimester as the findings did not show obvious safety warning signs. Nevertheless, recruiting larger numbers of participants and preferably with longitudinal monitoring is crucial to further strengthen the conclusion made earlier.

## Data Availability Statement

The raw data supporting the conclusions of this article will be made available by the authors, without undue reservation.

## Ethics Statement

The studies involving human participants were reviewed and approved by Medical Research and Ethics Committee (MREC) Malaysia. Written informed consent to participate in this study was provided by the participants' legal guardian/next of kin.

## Author Contributions

All authors listed have made a substantial, direct, and intellectual contribution to the work and approved it for publication.

## Conflict of Interest

The authors declare that the research was conducted in the absence of any commercial or financial relationships that could be construed as a potential conflict of interest.

## Publisher's Note

All claims expressed in this article are solely those of the authors and do not necessarily represent those of their affiliated organizations, or those of the publisher, the editors and the reviewers. Any product that may be evaluated in this article, or claim that may be made by its manufacturer, is not guaranteed or endorsed by the publisher.
